# Martial arts striking sports prehabilitation programme (MASS-12): Jump higher, move safer, and feel better

**DOI:** 10.1016/j.jsampl.2025.100134

**Published:** 2026-01-17

**Authors:** Rowan Wilson, James Brighton, Colette Christiansen, Matthew Hughes, Wendi Bacon

**Affiliations:** 1 PhysioFit Cambridge, United Kingdom; 2 School of Psychology & Life Sciences, Canterbury Christ Church University, United Kingdom; 3 School of Mathematics & Statistics, The Open University, United Kingdom; 4 Milton Keynes University Hospital, United Kingdom; 5 School of Life, Health & Chemical Sciences, The Open University, United Kingdom

**Keywords:** Prehabilitation, Injury prevention, Dynamic knee valgus, Recreational sport, Practitioner-informed

## Abstract

**Background:**

Martial arts involve cutting, jumping and landing movements known to increase injury risk. Martial artists frequently sustain injuries. Recreational martial arts have yet to implement an evidence-informed prehabilitation programme to prevent these injuries. Here, we evaluate the Martial Arts Striking Sports prehabilitation programme (MASS-12) in a recreational setting.

**Methods:**

Three traditional martial arts warm-ups and two MASS-12 warm-ups were delivered over 2 weeks as a recreational Jiu Jitsu club hall. A coach and seven athletes (five male, two female), ranging in experience from white belt to brown belt, participated. Main Outcome Measures were the Frontal plane projection angle (FPPA), a measure of dynamic knee valgus and Single leg vertical hops (SLVH), a measure of performance.

**Results:**

Over half of recreational athletes reported previous, serious lower limb injuries. Athlete FFPA and SLVH significantly improved (−11.9° to −1.93°, 25.2 vs 27.5 cm, respectively) after the MASS-12. Athletes appreciated the increased feedback and sense of preparedness. The coach found it easy to teach and perceived improvements in performance.

**Conclusions:**

The MASS-12 is easy for coaches to implement; improves lower limb alignment; and is accepted by athletes. A significant improvement on current practices, the MASS-12 should be widely implemented in recreational martial arts.

**Contribution of the paper**

• Evidence-based prehabilitation programmes reduce injuries, particularly in female athletes. Recreational martial arts currently have no such programme.
• The MASS-12 uniquely applies injury prevention science to a recreational martial arts environment, improving lower limb alignment and engaging coaches and athletes without impacting performance.
• The MASS-12 is ready for implementation across martial arts striking sports, providing an informed baseline warm-up in an otherwise unregulated field.

## 1 Background

Most martial arts injuries occur during training rather than competition [1]. These disciplines span diverse movement styles, from grappling which involves close-contact, throws, falls, and locks (Judo, Jiu Jitsu, etc.) to striking (Tae-Kwon-Do, boxing etc.). Training includes ‘high risk’ movements such as cutting, jumping and landing [2]. Many traditional styles require barefoot practice without orthotic support – often on inconsistent surfaces like mats or school hall floors. Injury rates are high, especially among experienced athletes [3].

Prehabilitation is a proactive, evidence-informed strategy to prevent injury, often used as a warm-up. Programmes like FIFA 11+ reduced football injuries by up to 70 % [4], including among senior, recreational players [5]. Despite its success, FIFA 11+ is not widely adopted at the recreational level, relying on coach and athlete engagement [6].

Unlike football, martial arts are largely unregulated; teaching often requires only a black belt, with minimal coaching or biomechanics training. The content of martial arts warm-ups is undocumented, and no standard prehabilitation programme exists. Martial arts attract diverse participants across age and ability. The IPPON programme, developed for Judo, targets advanced athletes with skills like walking handstands [7]—unsuitable for recreational populations. IPPON also showed no reduction in injury frequency or severity [8]. Most prehabilitation programmes target young, elite athletes; their impact on recreational practitioners is often unclear.

The first evidence-based programme for Martial Arts Striking Sports, the MASS-12^9^, remains untested. This study compares MASS-12 with a traditional martial arts warm-up (TMAW) in a recreational setting. We hypothesised that MASS-12 would improve knee alignment during squat and jump tasks without compromising performance. We assessed movement mechanics (as injury risk indicators) and athlete/coach experiences (to gauge adherence). This is also the first study to quantitatively and qualitatively characterise a TMAW.

## 2 Methods

### 2.1 Ethical approval

Researchers obtained ethical approval from the university Human Research Ethics Committee (HREC: 4359) before engaging with a recreational martial arts club (RMC) practicing a Japanese Jiu Jitsu style incorporating strikes, throws, locks, and groundwork. Participant information and consent forms for coach and athletes are in Appendix A.

### 2.2 Coach participation

The RMC head coach agreed to participate, with a £150 donation covering hall fees. Study results were shared with coaches pre-publication. Due to personal ties with the research team, the head coach nominated a junior coach (self-identified man, <1 year coaching experience) unknown to the team. After delivering the TMAW in the first three sessions, he practiced the MASS-12 with authors 1 and 5 for 2 h, followed by 2 h of independent study. He delivered the MASS-12 in the final two sessions, observed and filmed by authors 1 and 5, who confirmed accurate delivery.

### 2.3 Study design

Athletes were informed via club social media and briefed in person before consenting. Data was collected over five sessions across three weeks (Fig. S1). Sessions began with a warm-up (TMAW for the first three, MASS-12 for the last two), followed by individual athlete evaluations. MASS-12 content is described elsewhere [9] and summarised in Fig. S2, while TMAW content was analysed in this study. Outcomes were compared for athletes attending >1 TMAW session (to assess learning effects) and athletes attending both TMAW and MASS-12 (Fig. S3). Participants ranged in experience from white belt (<3 months experience) to brown belt (3+ years) (Table S1). Due to low numbers, experience, sex, and injury history were excluded from statistical analyses.

### 2.4 Data collection

Warm-ups were filmed. Athletes then performed perform two tests: a single leg squat (SLS) and single leg vertical hop (SLVH), assessing movement quality and performance. Tests were filmed in frontal and sagittal planes. Prompts included:

1. Which leg would you kick a ball with? Stand on that leg.
2. Squat as low as you can, 3 times
3. Reach up as high as you can and touch your hand to the wall
4. Hop as high as you can, and touch the wall as high as you can, 3 times

Qualitative data was collected by Author WB [cis-gendered woman, martial arts coach, MASS-12 co-creator] who asked, “What did you think of the warm-up today?”. This question was an open invitation for extended reflections on the lived embodied experiences of undertaking MASS-12 and was recorded using a Dictaphone.

Athletes then resumed training. Before leaving, athletes anonymously rated the statement: “The warm-up prepared me well for my training session today” on a Likert scale [10].

Although researchers did not ask about injuries or health history, athletes disclosed this verbally, and it was recorded.

### 2.5 Movement quality

**Fig. 1:**
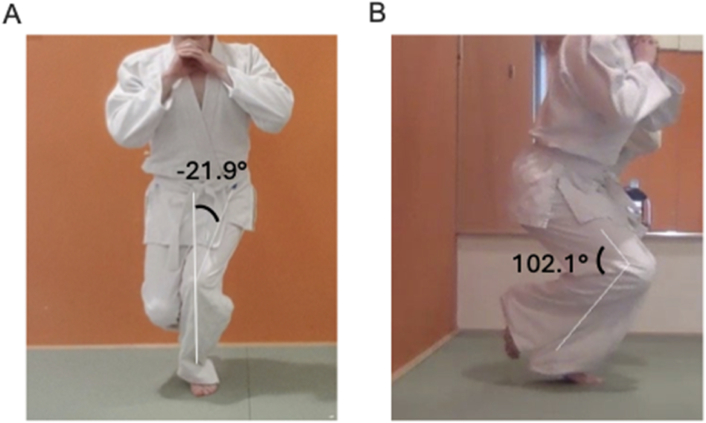
Angle measurements. A) FFPA angle and B) knee flexion angle exemplars.

Movement quality in SLS and SLVH was assessed using the frontal plane projection angle (FFPA), a 2D quantification of dynamic knee valgus [11], an established risk factor [12]; and the Qualitative Analysis of Single Leg Loading Score (QASLS), a checklist of movement impairments [13]. The FFPA is measured in degrees, while the QASLS is discrete, where a given point represents a problem with the mechanics (e.g., knee valgus) (Fig. 1A).

Still images at the lowest movement point were extracted from videos, yielding 516 images. These were randomised and blindly analysed by two physiotherapists (Authors 1 and 4), identifying anatomical landmarks (anterior-superior iliac spine (ASIS, the middle of the patella, and the midpoint between the medial and lateral malleolus in line). FFPA was measured using ImageJ [14] (Manufacturer: Wayne Rasband & contributors, National InstitutesHealth, USA; Version 1.53k). Triplicate videos were blindly scored using QASLS.

### 2.6 Performance

Performance was evaluated via SLVH height [15] and depth during SLS and SLVH. Depth was measured as knee flexion angles from sagittal stills, where measurements were taken along the centre of the thigh through the knee joint to the lateral or medial malleolus depending on camera angle (Fig. 1B).

### 2.7 Warm-up behaviors

To compare MASS-12 with TMAW content, warm-up videos were characterised by a martial arts practitioner (Author 5) and physiotherapist (Author 1) for physical and social behaviors, referencing prior studies (Table S1). Behaviors exhibited at any point during each minute were logged where both authors agreed. As warm-ups durations varied, data was expressed as percentage time per activity.

### 2.8 Thematic analysis

Participant responses (n_TMAW_ = 21, n_MASS-12_ = 12, total responses) to “What did you think of the warm-up today” were transcribed verbatim and analysed by Author 2 using Braun et al.’s [16] six-phase thematic analysis approach. Author 2 read each transcript twice and coded meaningful segments (Phase 1–2). Themes were checked with other authors, who independently analysed transcripts and developed themes (Phases 3–5). Conceptions were changed, merged or omitted and new categories were generated or restructures to reveal new relationships [17,18]. Final themes were negotiated to produce a coherent overview before write-up (Phase 6). Rather than seeking inter-rater consensus, this process aimed to develop plausible interpretations of participants' reflections, avoiding the pursuit of a singular ‘truth’ (see Smith and Sparkes [19]).

### 2.9 Data processing

All videos were scored. For image analysis, exercises were excluded if participants tapped down with an arm or leg, or if anatomical landmarks were obscured. All comparisons used averaged physiotherapist scores.

For TMAW vs TMAW comparisons, participants needed ≥2 successful repetitions in two TMAW sessions. For TMAW vs MASS-12 comparisons, participants needed ≥2 repetitions in one session of each type. Scores were averaged per participant per session, then compared across sessions.

### 2.10 Statistical analysis

Normality was assessed via a Shapiro–Wilk test, where *p* > 0.05 is needed for a pass. Paired t tests were used due to repeated measures. A two-sided paired t test assessed whether repeated testing (TMAW1 vs TMAW2) influenced movement or performance. A one-sided paired t test evaluated whether MASS-12 improved outcomes compared to TMAW. Enjoyability was measured using a 5-point Likert scale, with average scores compared for TMAW vs MASS-12. SLVH height was compared between one TMAW and one MASS-12 session using a two-sided paired t test. Warm-up behaviors were analysed descriptively due to small sample size (n_TMAW_ = 3 sessions, n_MASS-12_ = 2 sessions). Graphs were created using Graphpad Prism. The acceptance threshold was set at *p* < 0. Results are visualised in bar graphs (summarised in Table 1). Full statistical details are in Table S3.

**Table 1:** Statistical summary.

**Frontal Plane Projection Angle (FFPA)**				
**Repeated measures**	Warm-up	FFPA	n	p
SLS	TMAW1	−5.36°	6	ns
	TMAW2	−7.25°		
**Evaluate MASS-12**				
SLS	TMAW	−11.8°	8	0.008
	MASS-12	−1.93°		
SLVH: Take-off	TMAW	−2.38°	5	ns
	MASS-12	−0.867°		
SLVH: Landing	TMAW	4.62°	5	ns
	MASS-12	6.15°		
**Qualitative analysis of single leg loading score (QASLS)**				
**Repeated measures**	Warm-up	QASLS	n	p
SLS	TMAW1	7.07	7	0.041
	TMAW2	5.57		
SLVH	TMAW1	6.36	7	ns
	TMAW2	6		
**Evaluate MASS-12**				
SLS	TMAW	6.86	9	ns
	MASS-12	5.33		
SLVH	TMAW	5.42	9	ns
	MASS-12	5.17		
**Jump height with SLVH**	Warm-up	Jump height (cm)	n	p
**Repeated measures**	TMAW1	24.2	6	ns
	TMAW2	24.7		
**Evaluate MASS-12**	TMAW	25.2	8	0.032
	MASS-12	27.5		

## 3 Results

### 3.1 Impact on movement quality

**Fig. 2:**
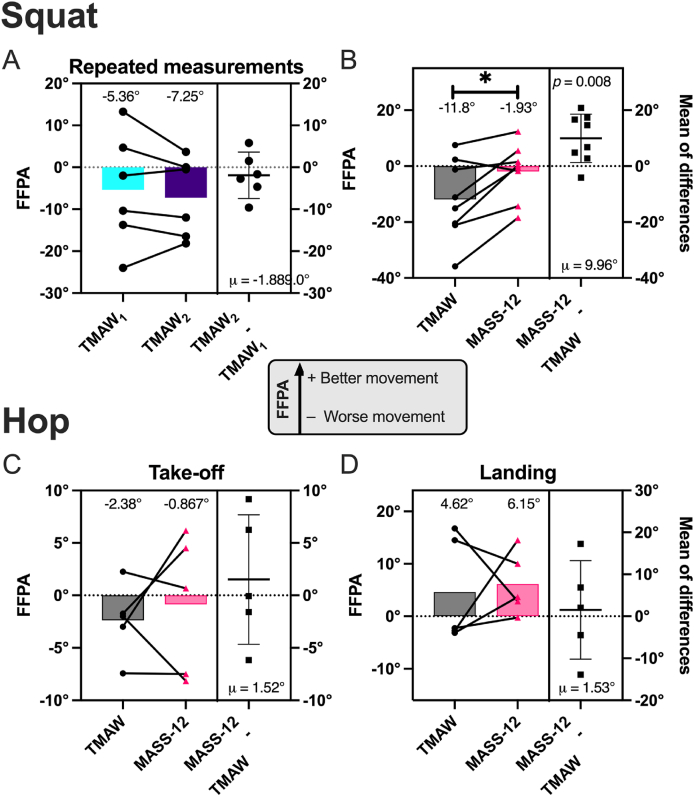
Dynamic knee valgus. A) FFPA was compared for SLS measured at two TMAW sessions; FFPA was compared between TMAW & MASS-12 sessions for B) SLS, C) SLVH take-off, and D) SLVH landing. Participants performed the exercise three times in each session, and the results were averaged to give a final value for the session.

Movement quality was quantified by FFPA and QASLS. Repeated TMAW sessions did not affect SLS FPPA (*n* = 6, μ_TMAW 1_ = −5.36°/*σ* = 13.4° vs μ_TMAW 2_ = −7.25°/*σ* = 9.4°, *p* = 0.42, *d* = −0.2, Fig. 2A). MASS-12 significantly improved SLS FPPA (*n* = 8, μ_TMAW_ = −11.8°/*σ* = 14.5° vs μ_MASS-12_ = −1.93°/*σ* = 10.0°, *p* = 0.008, *d* = 0.84, Fig. 2B). High SLVH exclusions prevented comparisons between TMAW sessions. TMAW values were already clinically safe (μ_Take-off_ = −2.38° & μ_Landing_ = 4.62°, Fig. 2C and D) prior to the MASS-12 intervention, which showed small, non-significant improvements.

**Fig. 3:**
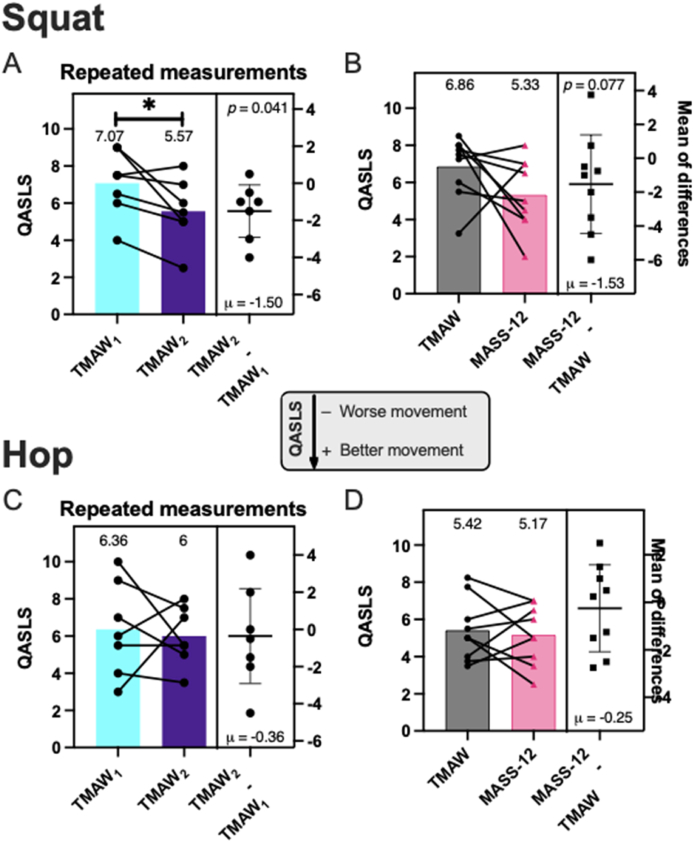
Overall movement quality. A) QASLS video evaluation was compared across TMAW sessions for SLS and C) SLVH. QASLS was compared between TMAW and MASS-12 sessions for B) SLS and D) SLVH.

Athletes improved SLS movement mechanics via QASLS with repetition (*n* = 7, μ_TMAW1_ = 7.07/*σ* = 1.77 vs μ_TMAW2_ = 5.57/*σ* = 1.74, *p* = 0.041, *d* = −0.99, Fig. 3A) and similar improvement after MASS-12 (*n* = 9, μ_TMAW_ = 6.86/*σ* = 1.67 vs μ_MASS-12_ = 5.33/*σ* = 1.92, *p* = 0.078, *d* = 0.84, Fig. 2B). No significant changes were found in jump quality after repeated TMAW sessions (*n* = 7, μ_TMAW1_ = 6.36/*σ* = 2.53 vs μ_TMAW2_ = 6/*σ* = 1.58, *p* = 0.774, *d* = 0.20, Fig. 3C) or after MASS-12 (*n* = 9, μ_TMAW_ = 5.42/*σ* = 1.68 vs μ_MASS-12_ = 5.17/*σ* = 1.60, *p* = 0.347, *d* = 0.15, Fig. 3D).

### 3.2 Impact on performance

**Fig. 4:**
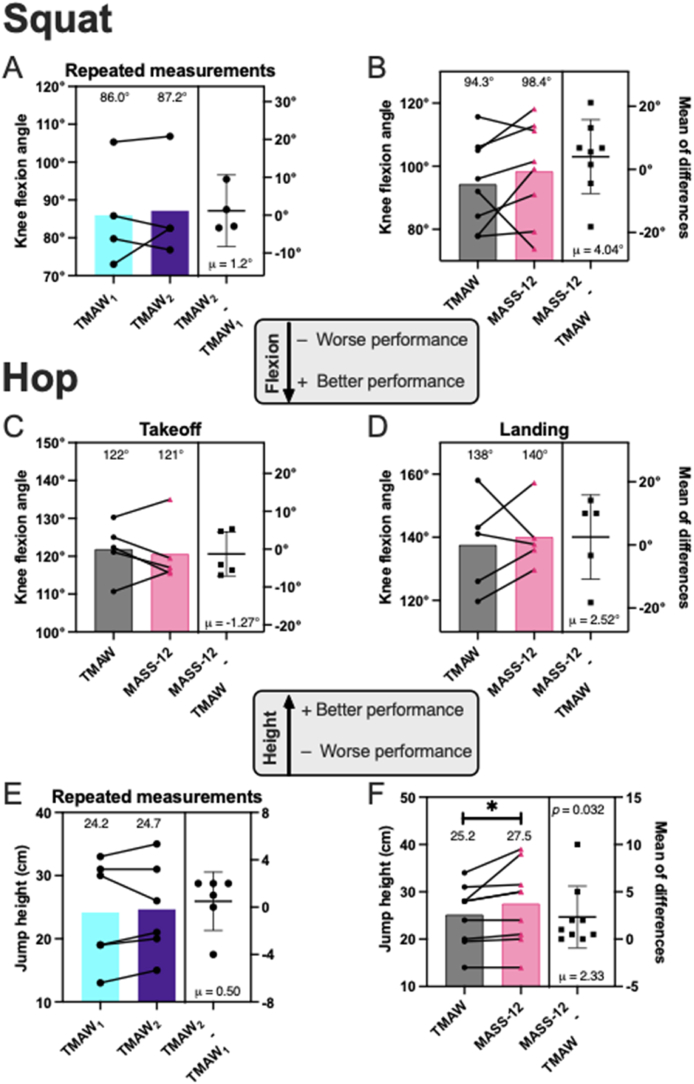
Performance indicators. Knee flexion during SLS was assessed from 2D sagittal images and compared A) within TMAW and B) between TMAW and MASS-12 sessions. SLVH knee flexion was compared for C) take-off and D) landing. The maximum distance gained in the SLVH was compared E) within TMAW and F) between TMAW and MASS-12 sessions.

We assessed whether MASS-12 compromised performance. No significant changes in knee flexion were found across sessions or warm-up types (Fig. 4A-D).

Jump height did not vary across repeated TMAW sessions (*n* = 6, μ_TMAW1_ = 24.2 cm *σ* = 8.2 cm vs μ_TMAW2_ = 24.7 cm/*σ* = 7.45 cm, *p* = 0.624, *d* = 0.08, Fig. 4E), but increased after MASS-12 (*n* = 8, μ_TMAW_ = 25.2 cm/*σ* = 6.33 cm vs μ_MASS-12_ = 27.5 cm/*σ* = 8.40 cm, *p* = 0.032, *d* = 0.31, Fig. 4F), indicating a performance gain without loss of jump quality.

### 3.3 Warm-up behaviors

**Fig. 5:**
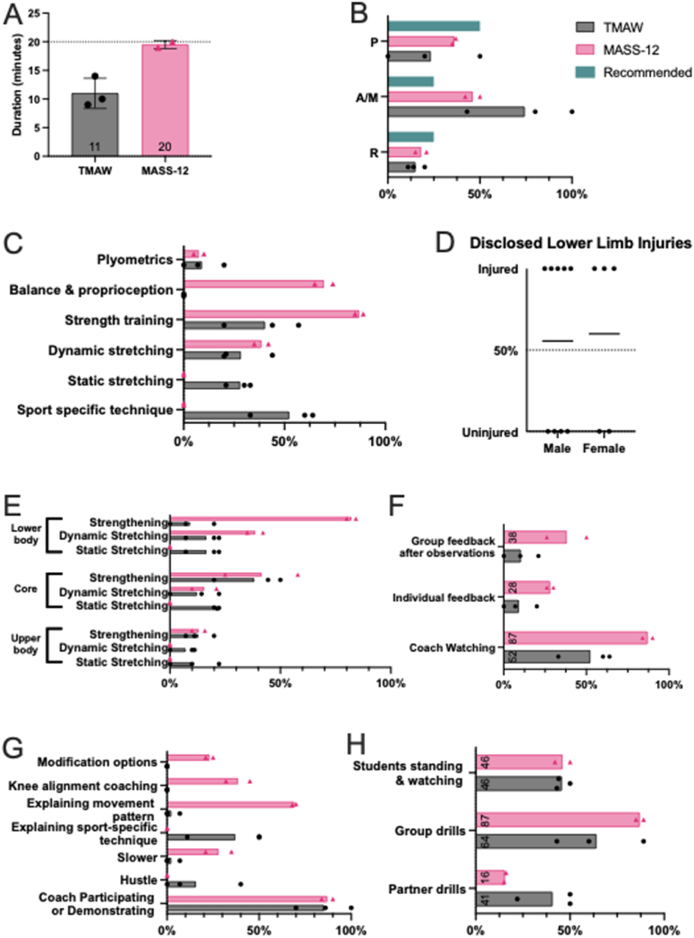
Warm-up behaviours. A) Length of warm-up in minutes, with an indicator at 20 min, the ideal warm-up duration [4]. B) Percentage of minutes containing exercises categorised as RAMP, with the recommended proportions indicated from [20]. C) Percentage of minutes containing categories of physical warm-up activities. D) Percentage of participants that disclosed concerning lower limb injuries, despite researchers not asking for this information. E) Percentage of minutes targeting upper body, core, or lower body in warm-up. F) Percentage of minutes wherein coach prepared and delivered feedback. G) Percentage of minutes wherein coach exhibited different types of coaching behaviours. H) Percentage of minutes wherein students exhibited different behaviours. Note that the percentages do not add up to 100 as more than one component can occur per minute.

TMAW averaged 11 min, while MASS-12 followed the recommended 20-min format (Fig. 5A). TMAW showed a 2-10-3 RAMP distribution, with less Potentiate (Fig. 5B), while MASS-12 aligned with the recommended 1-1-2 ratio [9,20].

TMAW emphasised sport-specific techniques, whereas MASS-12 included more strength and proprioception exercises (Fig. 5C). Athletes were not asked for their injury status, yet more than half disclosed major lower limb current or previous injuries/disorders that impacted their performance (Fig. 5D), prompting analysis by body region. TMAW favoured core static stretching, while MASS-12 targeted lower limbs (Fig. 5E).

MASS-12 coaching emphasised movement quality, with demonstrations followed by individual and group feedback (Fig. 5F). TMAW delivery lacked instructions on movement patterns, knee alignment, quality or modification - key components of prehabilitation (Fig. 5G). Group dynamics were similar across warm-ups, with no increase in idle time during MASS-12 despite its instructional demands (Fig. 5H)._under

### 3.4 Athlete & coach perception

**Fig. 6:**
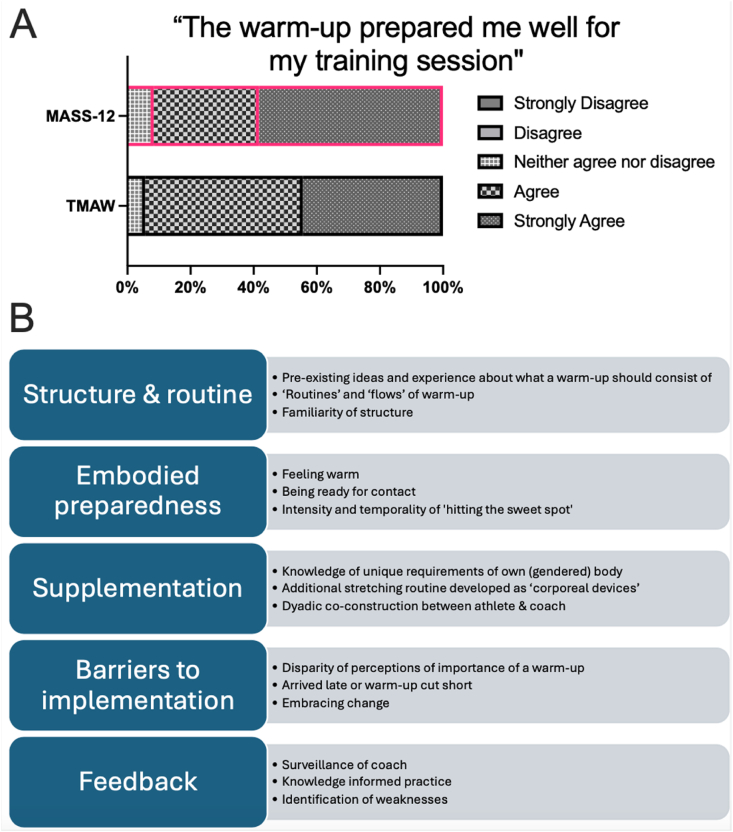
Athlete Experiences. A) Normalised Likert scale analysis to the question “The warm-up prepared me well for my training session” anonymously voted after each session. B) Summarised thematic analysis of “What did you think of the warm-up today?” open-ended prompt.

Quantitative ratings showed no difference in perceived preparedness (*n* = 30, μ_TMAW_ = 4.4 vs μ_MASS-12_ = 4.5, Fig. 6A). Thematic analyses revealed five key themes reflecting on general warmups and MASS-12 (Fig. 6B).

#### 3.4.1 Structure and routine

Athletes entered the MASS-12 with expectations of what a warm-up ‘should’ include. Experienced participants quickly recognised its evidence-informed design: “Very good structure - you can see that whoever did it really knew what they were doing, specifically for legs”. This created new movement patterns which challenged previous experiences: “It was a lot more technically difficult than we normally do. Normally, it’s like, stamina-wise, it’s quite difficult, but this was very technical, but this was good, it was good to test out new muscles, which we don’t normally do”. New learned routines were constructed which increased confidence that the warm-up facilitated the execution of more complex movements. “I liked the warm-up today, was a little bit more of the dynamic stretching, to get the same sort of motion you get in the throws, which are all very dynamic actions”. “I definitely feel a bit more stretchy than usual …. . it was a bit more yoga-esque”

#### 3.4.2 Embodied preparedness

Athletes drew on corporeal markers gauge MASS-12 effectiveness. Athletes used senses of thermo-perception including “deepness of breath”, “sweating”, and “muscular soreness” in articulating how the warm-up made them “feel warm” and ready for exercise. “It was nice and intense, hard-going, get a bit of a sweat on, so yeah, I felt warm by the end of it”. Athletes described a “sweet spot” in how their bodies felt prepared: “it got me properly warm … without being too exhausted to do anything”. Not being warm enough resulted in feeling under prepared, risking injury. Conversely, over-exertion in the warm-up could make athletes feel tired, also risking injury. “It’s enough that I’m not likely to injure myself later”. The progressively more intense nature of the warm-up allowed participants to adjust to increasing demands. Athletes also reported *feeling* that their bodies were worked in more challenging and diverse ways under the MASS-12 than the TMAW: “I think it’s just because of the warm-up. I was more stable because we did the stabilising, even though I feel more lactic acid in my leg, I just feel I can perform better”.

#### 3.4.3 Supplementation

Athletes routinely felt the need to add additional warm-up routines to supplement their own unique bodies. Athletes used previous experiential knowledge of muscular or anatomical imbalances and injury to undertake additional stretching routines: “It was good, but I end up doing more stretching off to the side, but that’s because of personal reasons … and injury”. Athletes acted as ‘*corporeal devices’* [21] where in and through their bodies they learn, regulate and make sense of warming up. One athlete noted that the MASS-12 helped expose anatomical weakness, ”it made me think a lot about my leg strength, because at the end of that my legs are iron, they’re sore, they’re tired”. Athletes recognised the importance of athlete-coach relationships, particularly coaches understanding each (gendered) athlete body. Here, athletes valued coach feedback tailored to individual needs. Athletes and coaches co-constructed regimens which supplemented the warm-up. The MASS-12’s structured approach reduced the need for supplementation.

#### 3.4.4 Barriers to implementation

Full benefits of the MASS-12 required complete participation, but adherence was impacted by time commitments and the importance placed on a warm-up. Athletes admitted that they routinely arrived late, cut warm ups short or just did their own thing. This resulted in them missing important parts of the routine requiring them to “push harder through the rest of the warm up”. Some athletes embraced the structure of the MASS-12, while others resisted: “it definitely felt different, and from what I know of martial artists, they can be strict with their routine, so it’s quite, what are they preparing us for today? Parkour, ballet?”. Such barriers, typical in amateur sport, threaten MASS-12 implementation.

#### 3.4.5 Feedback

Across themes, athletes emphasised the value of expert-led warm-ups. “I like the fact that they asked for some feedback, if people have any injuries, if people want to do some more. I got some feedback on my rolls too”. Athletes were happy to let the coaches take control of their bodies in the warm-up because they could see that expert knowledge was informing warm-up practices which further engrained confidence in the coach’s expertise for the session ahead: “It was good, it was different … I feel I can perform better”. The MASS-12 also revealed athlete weaknesses, prompting targeted coaching feedback and evolving a knowledge informed approach to coaching.

## 4 Conclusions

Calls for a martial arts-specific, evidence-informed warm-up date back to 2001 [22]. However, implementation at a recreational level depends on coach and athlete buy-in [6]. This study pioneers a holistic approach, evaluating physical outcomes with social reception of a rehabilitation programme. Mixed methods allowed coach and athletes to both experience and reflect on traditional and modern warm-ups.

Unlike typical 6–12 week prehabilitation trials, MASS-12 significantly improved knee alignment in a single session. The FPPA - a proxy for knee valgus [23] which is a risk factor for injuries [24,25] - improved from 11.9° to −1.93° during SLS, shifting from a clinically risky to a clinically safe movement. FFPAs as high as −5.2° have predicted knee injury risk [25]. In high impact sports involving explosive weight shifting and pivoting, such improvements are linked to reduced injuries [[26], [27], [28], [29]]. Improved mechanics did not impact performance.

Improving alignment after a single MASS-12 session was surprising, and can be interpreted in two ways: first, that TMAWs are so insufficient for the demands of martial arts that any informed intervention – be it based on proprioception, strength training, or both - would yield improvements; and/or second, that martial arts athletes are adept at acquiring movement patterns, supported by literature on martial arts’ benefits to balance, coordination and cognition [30].

TMAW focused on static stretching and sport-specific drills, reflecting coaching expertise in sport but not biomechanics - a common gap among recreational coaches [31,32]. Dynamic stretching is favoured for injury prevention and performance [20]. TMAW’s emphasis on upper body movements likely reflects this sporting emphasis. The user-friendly MASS-12 bridged this knowledge gap, emphasising lower body techniques - relevant given the high rate of disclosed lower limb injuries, consistent with martial arts injury trends [33]. TMAW included more partner drills. While emphasising contact early aligns with contact-oriented training, problematic contact is a barrier to women’s participation [34]. In recreational martial arts - where all genders and experience levels train together - optimal contact levels during warm-ups remains unclear.

### 4.1 Coaching feedback

A key principle in MASS-12 – and prehabilitation broadly - is prioritising quality over quantity [12]. Martial arts instruction typically involves coach participation, limiting athlete observation and feedback. Athletes in this study reported limited feedback during TMAWs. MASS-12’s emphasis on movement quality led to coach-perceived performance improvements, paired with a reflection on the importance of feedback over coach participation. Previous study showed that coach feedback and instruction is higher during skills practice than warm-up or sparring, where their own participation increases [35]. Martial arts culture values technical feedback [36]. Verbal cues like “Land softly” [37] can improve landing mechanics. Self-reflection (videos) and expert feedback also improve mechanics [38]. Despite the value of expert feedback, martial arts coaches typically rate their expertise by their own athletic ability – a trait athletes rate as low importance [39]. MASS-12 unexpectedly empowered the coach to provide anatomical feedback, and re-evaluate their coaching role. MASS-12 implementation may, therefore, change the perception of coach athletic performance as a key value indicator.

### 4.2 Athlete experience

We uniquely included athlete perspectives, addressing a gap in intervention studies that limits uptake [32]. Athletes revealed a disconnect between their understanding of warm-up purpose and evidence-based practices, highlighting a target for intervention. Martial arts’ emphasis on tradition and loyalty to styles and instructors [40] may exacerbate common barriers to implementation. Embracing change can be challenging for routine-driven athletes, while improved biomechanical understanding may facilitate inclusion of athlete-specific movements to increase buy-in for implementation across populations. Positive feedback on MASS-12 structure, flow, and flexibility will further support implementation – especially as it was co-developed by martial artists and researchers.

### 4.3 Recreational setting

Recreational athletes vary widely in age, body type, and athleticism, while researchers often favor homogeneous groups. Uniquely, we measured significant impact from MASS-12 in this heterogeneous recreational population. The coach learned MASS-12 quickly and delivered it accurately, confirming its feasibility for recreational settings.

### 4.4 Limitations

Sporadic attendance led to small samples. The unblinded design may have influenced athlete motivation - athletes may view a ‘new’ warm-up positively due to novelty or clinical framing, while others may see it as external interference in established routines.

Overall movement quality did not improve, likely due to the single-session limitation. Longer trials (6–8 weeks) may yield better results, consistent with other prehabilitation studies [4]. Knee valgus was measured as a proxy for injury risk. Future studies should include long-term injury surveillance, though challenging in recreational settings.

SLVH data was limited due to athlete rotation and anatomical landmark obscuration.

Although MASS-12 was designed for striking arts, it was trialled in a mixed striking/grappling setting. High-risk movement mechanics are even more severe in a grappling setting where pivoting and stop-start movements occur with not one athlete’s weight, but two. Life the FIFA-11’s adaptation to basketball [41], MASS-12 may be applicable across martial arts styles.

The RMC was originally chosen due to its proportionally high female participation rates, however female attendance was sporadic and few completed the study. Female participation was low, although in line with nationwide female participation rates [42]. For this reason, we added an extra evaluation session (the 5th session) specifically to ensure study completion by both female participants. LGBTQ and racial diversity was high for the numbers and region.

## 5 Summary

The MASS-12 is an evidence-based prehabilitation programme that improves lower limb biomechanics, shows coach usability is well-received by athletes. While further research is needed to tailor it to specific martial arts styles and assess long-term outcomes, the MASS-12 is a substantial improvement over current martial arts warm-ups and is ready for wider implementation.

## Data availability

Participant information and consent forms are available in Appendix A. All data, including quality control assessments and pre-processing, is available in the Supplemental Information.

All images containing angle measurements from physiotherapists are available on request, due to the large (>1000 images) size of the dataset. The GraphPad Prism file containing all data necessary for reproducing both statistical analyses and figures is also available on request.

## Contributorship

WB planned, conducted, and reported the work, as well as secured funding and put the team together. RW co-created the MASS-12 with WB, planned and conducted the work, as well as edited the final manuscript. MH evaluated images and videos. JB evaluated the qualitative data, while CC performed quantitative analysis. JB, CC, and RW critically reviewed the manuscript. All authors have read and approved the final version of this manuscript, and agree with the presentation of the authors.

## Declaration of generative AI and AI-assisted technologies in the writing process

During the preparation of this work the authors used Microsoft Copilot in order to meet the word count limit. After using this tool/service, the authors reviewed and edited the content and take full responsibility for the content of the publication.

## Funding

This work was funded, in part, by the Private Physiotherapy Education Foundation (Award 366) and The Open University Impact Acceleration fund, for which we are grateful. The funders had no other involvement in this study.

## Declaration of competing interest

The authors declare that they have no known competing financial interests or personal relationships that could have appeared to influence the work reported in this paper.
