# Supplementary material for: Martial arts striking sports prehabilitation programme (MASS-12): Jump higher, move safer, and feel better
Source: JSAMS Plus. 2026 Jan 17;7:100134. doi: 10.1016/j.jsampl.2025.100134 (PMC13227172; doi:10.1016/j.jsampl.2025.100134)

# Supplemental Information

1. Appendix A: Coach and Athlete PI & Consent forms
2. Table S1: Participant information summary
3. Table S2: Warm-up behaviors
4. Figure S1: Study timeline
5. Figure S2: MASS-12 Programme
6. Figure S3: Study design

### Table S1: Participant information summary

| **Belts** | **Minimum training experience per belt** | **Age** | **Sex** |
| --- | --- | --- | --- |
| 2 White | NA | 20 - 40 years | 2F; 7M |
| 1 Orange | 6 months |  |  |
| 1 Green | 9 months |  |  |
| 2 Purple | 15 months |  |  |
| 2 Light Blue | 21 months |  |  |
| 1 Brown | 39 months |  |  |

### Table S2: Warm-up behaviors

| **Type** | **Category** | **Activity** | **Rationale** |
| --- | --- | --- | --- |
| Physical | RAMP | Raise tissue temperature | ^20^ |
| Physical | RAMP | Activate & Mobilise | ^20^ |
| Physical | RAMP | Potentiate | ^20^ |
| Physical | Sport specific technique | Sport specific technique | Renamed from ‘Skills practice’  to distinguish between martial skills and exercise movements  ^35^ |
| Physical | Static stretching | Static Stretching* | ^20^ |
| Physical | Static stretching | Dynamic Stretching* | ^20^ |
| Physical | Static stretching | Strength Training* | ^20^ |
| Physical | Balance & proprioception | Balance & proprioception | ^27^ |
| Physical | Plyometrics | Plyometrics | ^20^ |
| Social | Pace | Students standing & watching | Renamed from ‘Instruction’ ^35^  Minimal standing and watching is needed for an effective warm-up |
| Social | Organisation | Partner drills | Physical contact in warm-up sets up comfort for partner training. |
| Social | Organisation | Group drills | Group drills embed teamwork and community. |
| Social | Pace | Hustle | ^35^ |
| Social | Pace | Slower | Created as an alternative to ‘Hustle’ |
| Social | Explanation | Explaining sport-specific technique | Subdivided ‘Instruction’ category ^35^ to distinguish between martial skills and exercise movements |
| Social | Explanation | Explaining exercise or movement pattern | Includes technical points and safety |
| Social | Coaching_Activity | Coach Participating / Demonstrating | Renamed from ‘Activity b’ ^35^ |
| Social | Coaching_Activity | Coach Watching | Subcategory of ‘Activity a’ ^35^ |
| Social | Feedback | Individual feedback | Distinguished from feedback categories ^35^ |
| Social | Feedback | Group feedback after observations | Subcategory of ‘Activity a’ ^35^ |
| Social | Explanation | Knee alignment coaching | Critical to lower limb injury prevention ^27^ |
| Social | Explanation | Modification options | A measure of flexibility in delivering a warm-up across multiple bodies |

*Static stretching, dynamic stretching, and strengthening were subdivided into upper body, lower body, and spinal/core to characterise body part emphasis

### Table S3: Statistical AnalysisFigure S1: Study timeline.
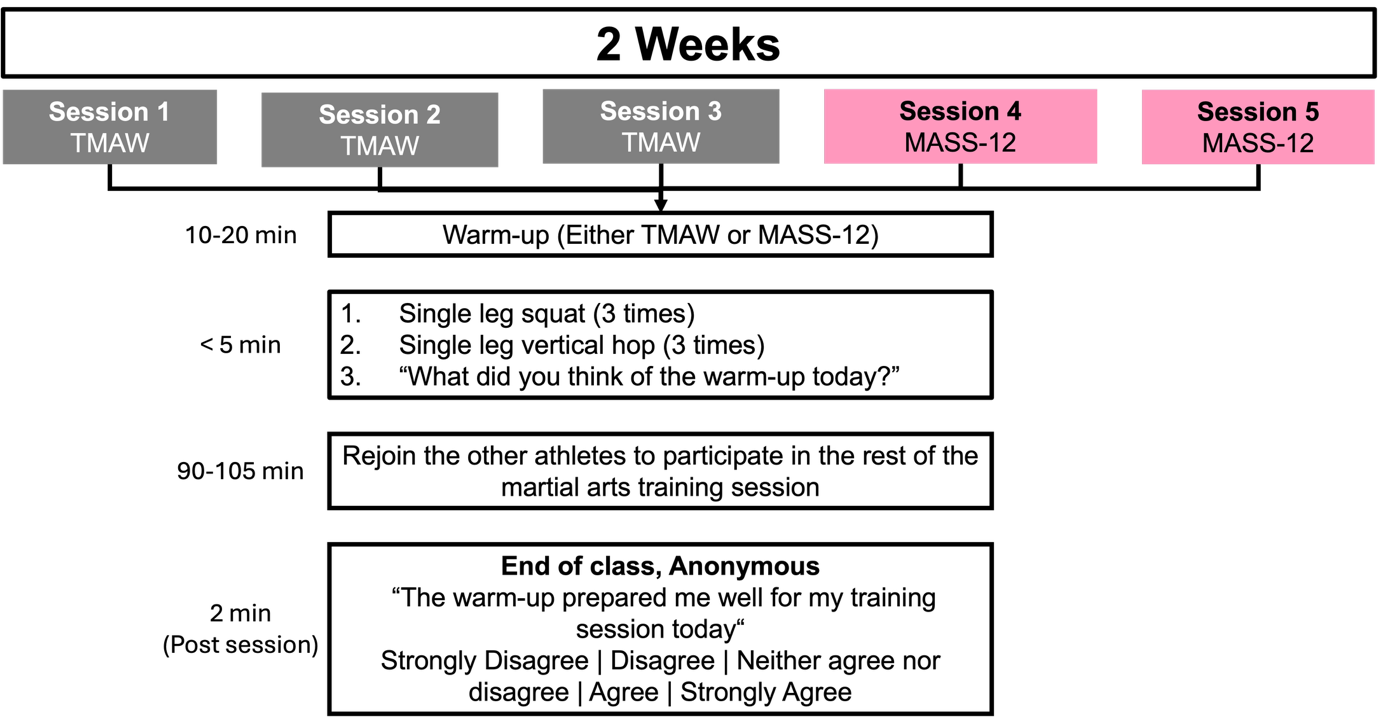


### Figure S2: MASS-12 contents. Reprinted with permission^9^


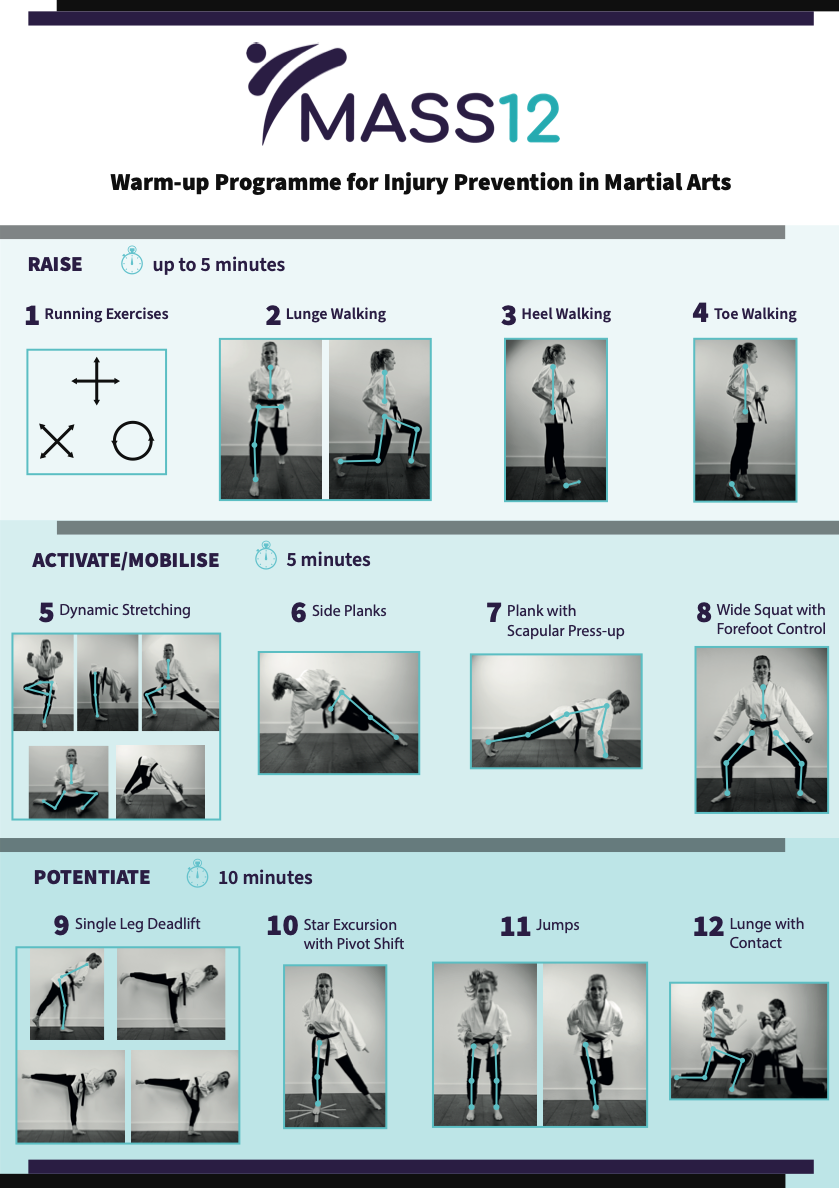


### Figure S3: Study design


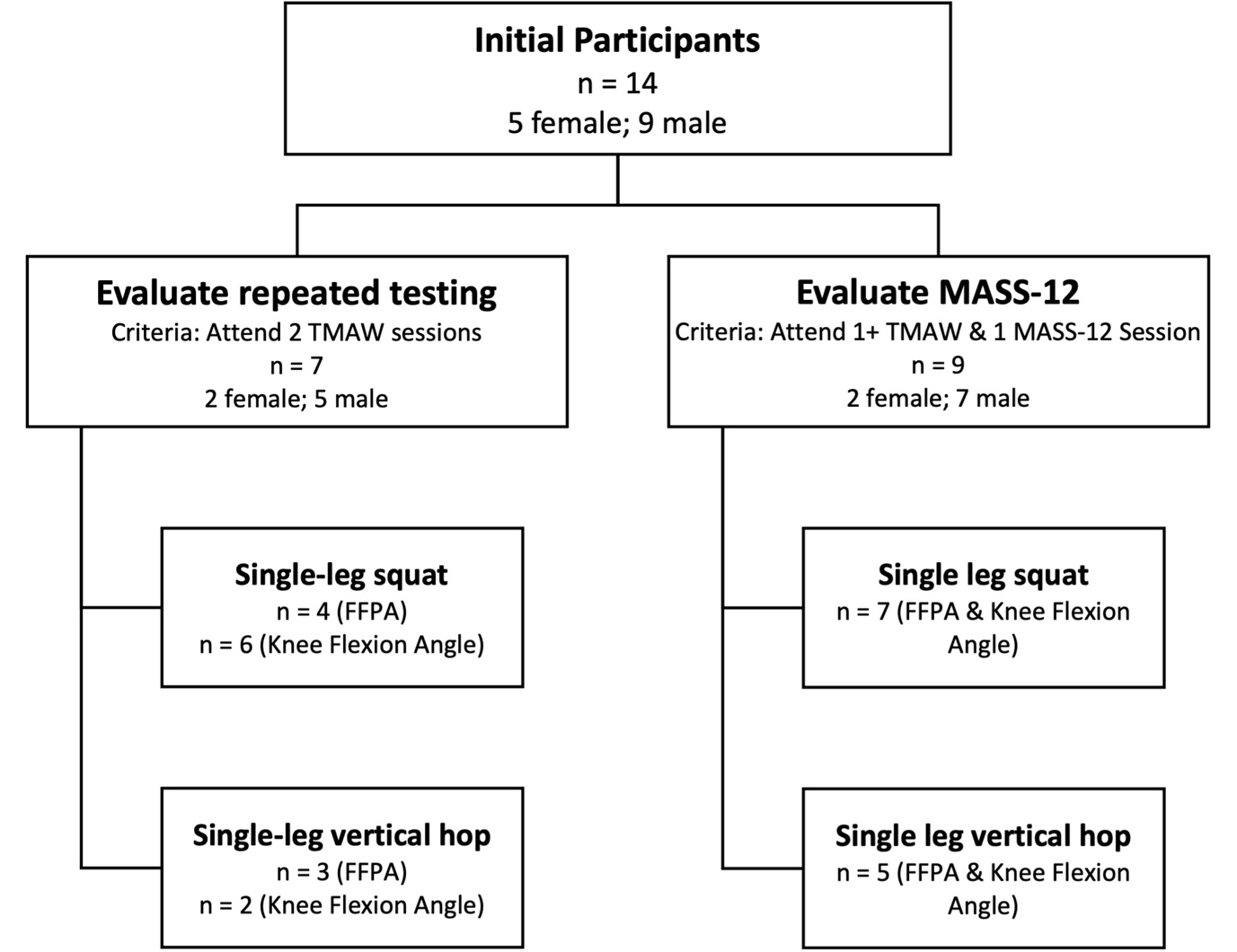

Supplement: Multimedia component 2 [file mmc2.docx]
