# Supplementary material for: Martial arts striking sports prehabilitation programme (MASS-12): Jump higher, move safer, and feel better
Source: JSAMS Plus. 2026 Jan 17;7:100134. doi: 10.1016/j.jsampl.2025.100134 (PMC13227172; doi:10.1016/j.jsampl.2025.100134)
Supplement: Multimedia component 3 [file mmc3.pdf]

CONSENT FORM

INFORMED CONSENT FOR EVALUATION OF MARTIAL ARTS WARM-UPS

Wendi Bacon, Lecturer in Health Sciences

Please highlight your choice by checking inside the appropriate box.

1. Taking part in the study

I have read and understood the information sheet for the following study: **Evaluation of Martial Arts Warm-ups**, or it has been read to me. I have been able to ask questions about my participation and my questions have been answered to my satisfaction.	YES ☐	NO ☐
I consent voluntarily to be a participant in this study and understand that I can refuse to answer questions I am not comfortable with and I can withdraw from the study at any time by contacting Wendi Bacon (wendi.bacon@open.ac.uk) up until data has been anonymised (five weeks after the first class of data collection) without having to give a reason.	YES ☐	NO ☐
I understand that taking part in the study involves video recording of me instructing two traditional warm-ups and a taught warm-up; working with researchers to learn to teach a new warm-up; and attending a coaching round-table to discuss results and feedback.	YES ☐	NO ☐
I understand that taking part in this study has no more risk than instructing a standard martial arts class. I understand and accept that taking part in the study, as it involves exercise, has some risk.	YES ☐	NO ☐

2. Use of the information in the study

I understand that information I provide will be used for (anonymous) feedback for coaches, journal articles, social media and coaching resources.	YES ☐	NO ☐
I understand that personal information collected about me that can identify me, such as my name, will not be shared beyond the study team.	YES ☐	NO ☐
I understand that my data will be transferred immediately (within 24 hours) to a password protected computer and server, anonymised within four weeks, and stored for up to ten years.	YES ☐	NO ☐
I agree to being quoted anonymously.	YES ☐	NO ☐

3. Future use and reuse of the information by others

I give permission for the anonymised video recordings, meeting recordings, and learning notes generated in this study to be deposited in a specialist data centre after it has been anonymised, so it can be used for future research and learning. Consent forms will be stored securely and digitally encrypted, while originals will be destroyed via shredding.	YES ☐	NO ☐

4. Signatures

**Name of participant**<br>**[in CAPITALS]**	**Signature**	**Date**
___________________________	_______________________	_________

For participants unable to sign their name, please mark the box instead of signing ☐

This research project has been reviewed by, and received a favourable opinion from, The Open University Human Research Ethics Committee – HREC reference number: 4359

RESEARCH STUDY PARTICIPANT INFORMATION SHEET

Coaches

CONTACT DETAILS

Principal Investigator: Wendi Bacon, wendi.bacon@open.ac.uk
Independent contact: Francesco Crea, francesco.crea@open.ac.uk

INVITATION

You are being invited to take part in a research study. Before you decide whether to take part, it is important for you to understand why the research is being done and what it will involve. Please take time to read the following information carefully. You have been chosen as one of the coaches of the Cambridge University Jiu Jitsu club, which provides sufficient number of students and retainment from class to class for this study.

GENERAL INFORMATION

The purpose of this research is to test the impact of a warm-up on athletic performance in martial arts in collaboration with PhysioFit, Cambridge. We are partially funded by the Private Physiotherapy Educational Foundation (https://ppef.org.uk) as well as an Impact Acceleration funding from the Higher Education Innovation Fund.

This research project has been reviewed by, and received a favourable opinion, from The Open University Human Research Ethics Committee – HREC reference number: 4359.

WHAT WILL I BE ASKED TO DO IF I AGREE TO TAKE PART?

This study will take place across three concurrent classes.

- To participate, you will plan three sessions, occurring on the same day of the week three times, that will put an emphasis on the striking (kicking, punching, boxing, elbows, knees, etc.) elements of your martial arts.
- You will communicate with your club in advance of the session that you will be participating in a research study on martial arts, and they are encouraged to come to all three classes to allow for matched data, if they wish to participate. You will let them know that the researchers are donating funds to cover the cost of the hall for these three sessions.
- You will run your normal warm-up as part of a class in the first two sessions. These warm-ups will be **recorded.**
- The researchers will explain that we are running a study, and ask willing students to fill out appropriate paperwork to participate in the study.
- You will then be trained in a Face-to-Face meeting by the researchers (Dr Bacon and Physiotherapist Rowan Wilson) on how to perform a distinct warm-up protocol.
- You will be given a packet of images and brief text describing each exercise to study and practice.
- You will then do a run-through (either virtually or face-to-face, your choice) of teaching the protocol to the researchers to ensure correct teaching. The researchers will **record notes** on the process of teaching and learning this warm-up.
- The third session, you will run this new warm-up. This warm-up will be **recorded.**
- After the researchers perform data analysis of the study, you will attend a coaching round-table (face-to-face or virtually, your club's choice) alongside your fellow club instructors. The researchers will share the data, and collect your feedback and that of the rest of the club instructors. This meeting will be **recorded.**

It is up to you to decide whether or not to take part. If you do decide to take part, you will be given this information sheet to keep and be asked to sign a consent form. There are no increased risks to taking part than in your normal martial arts training and coaching.

Participation will improve our understanding of how warm-ups impact martial arts performance. We will share the data in a coaching roundtable to ensure that any findings are communicated to and directly benefit your club.

HOW WILL THE DATA I PROVIDE BE USED?

Video data, meeting recordings and researcher notes will be transferred to a secure Open University server, and shared only with the researchers working on this project. Names will be anonymised. Raw (anonymised) data will be retained for up to ten years. Signed consent forms will be stored safely in a locked OU office.

Results will be first disseminated to club instructors and may later published in a peer-reviewed journal, shared on social media, and/or distributed as coaching resources across clubs. Any included images will have face or other identifying features cropped or blurred.

YOUR RIGHT TO WITHDRAW FROM THE STUDY

You have the right to withdraw from the study at any time during your participation by emailing wendi.bacon@open.ac.uk. You cannot withdraw once matched data has been anonymised, which will occur approximately five weeks after data collection.

HOW DO I AGREE TO TAKE PART?

To participate, please sign and return the consent form to the researchers, who will arrange dates.

THANK YOU

Thank you so much for reading this information sheet and participating in our study.

To be notified of any results to this study or others, or to find out about future studies in which you could participate, you can subscribe to our mailing list by contacting wendi.bacon@open.ac.uk

---

DATA PROTECTION

The Open University is the Data Controller for the personal data that you provide.

The lawful reason for processing your data will be that conducting academic research is part of The Open University's public task. The consent we request from you relates to ethical considerations.

No personal data (name) will be shared outside of the OU and data will be anonymised within 5 weeks of collection.

You have a number of rights as a data subject:

- To request a copy of the personal data we have about you
- To rectify any personal data which is inaccurate or incomplete
- To restrict the processing of your data
- To receive a copy of your data in an easily transferrable format (if relevant)
- To erase your data
- To object to us processing your data

If you are concerned about the way we have processed your personal information, you can contact the Information Commissioner's Office (ICO). Please visit the ICO's website for further details.

CONSENT FORM

INFORMED CONSENT FOR EVALUATION OF MARTIAL ARTS WARM-UPS

Wendi Bacon, Lecturer in Health Sciences

Please highlight your choice by checking inside the appropriate box.

1. Taking part in the study

I have read and understood the information sheet for the following study: **Evaluation of Martial Arts Warm-ups**, or it has been read to me. I have been able to ask questions about my participation and my questions have been answered to my satisfaction.	YES ☐	NO ☐
I consent voluntarily to be a participant in this study and understand that I can refuse to answer questions I am not comfortable with and I can withdraw from the study at any time by contacting Wendi Bacon (wendi.bacon@open.ac.uk) up until data has been anonymised (five weeks from the first data collection), without having to give a reason.	YES ☐	NO ☐
I understand that taking part in the study involves video recording of me in class performing a warm-up across three weeks, video recording of me doing two exercises after the warm-up each week, recording of my name and biological sex, voice recording of my experience performing the warm-up, and a rating of how well the warm-up helped me train.	YES ☐	NO ☐
I understand that taking part in this warm-up and assessment has no more risk than a standard martial arts class. I understand and accept that taking part in the study, as it involves exercise, has some risk.	YES ☐	NO ☐

2. Use of the information in the study

I understand that information I provide will be used for (anonymous) feedback for coaches, journal articles, social media and coaching resources.	YES ☐	NO ☐
I understand that personal information collected about me that can identify me, such as my name, will not be shared beyond the study team.	YES ☐	NO ☐
I understand that my data will be transferred immediately (within 72 hours) to a password protected computer and server, anonymised within five weeks, and stored for up to ten years.	YES ☐	NO ☐
I agree to being quoted anonymously.	YES ☐	NO ☐

3. Future use and reuse of the information by others

I give permission for the anonymised video recordings and voice recordings that I provide to be deposited in a specialist data centre after it has been anonymised, so it can be used for future research and learning. Consent forms will be stored securely and digitally encrypted, while originals will be destroyed via shredding.	YES ☐	NO ☐

4. Signatures

Name of participant [in CAPITALS]	Signature	Date
______________________	______________________	__________

For participants unable to sign their name, please mark the box instead of signing ☐

This research project has been reviewed by, and received a favourable opinion from, The Open University Human Research Ethics Committee – HREC reference number: 4359

RESEARCH STUDY PARTICIPANT INFORMATION SHEET

CONTACT DETAILS

Principal Investigator: Wendi Bacon, wendi.bacon@open.ac.uk
Independent contact: Francesco Crea, francesco.crea@open.ac.uk

INVITATION

You are being invited to take part in a research study. Before you decide whether to take part, it is important for you to understand why the research is being done and what it will involve. Please take time to read the following information carefully. You and your classmates have been chosen as you are martial artist students of various levels. Whether you choose to participate or not will in not impact your martial arts training, as we are not affiliated with The Jitsu Federation.

GENERAL INFORMATION

The purpose of this research is to test the impact of a warm-up on athletic performance in martial arts in collaboration with PhysioFit, Cambridge. We are partially funded by the Private Physiotherapy Educational Foundation (https://ppef.org.uk) as well as an Impact Acceleration funding from the Higher Education Innovation Fund.

This research project has been reviewed by, and received a favourable opinion, from The Open University Human Research Ethics Committee – HREC reference number: 4359.

WHAT WILL I BE ASKED TO DO IF I AGREE TO TAKE PART?

This study will take place across three classes, held one week apart.

- To participate, you will be **recorded** during the standard warm-up as part of normal class in the first two weeks, and **recorded** again performing a different warm-up in the third week.
- Each week, you will then be asked your **name** (for ensuring data is matched across each week) and in the first week, we will ask for your **biological sex** (for anatomical classification only).
- You will then perform two exercises – a single leg squat and a single leg hop – while being **recorded** individually. You will have three attempts at each exercise, each week.
- We will then ask you what you thought of the warm-up, and we will **record** your answer.

This process will take roughly 5 minutes.

- Finally, at the end of class, you will anonymously **rate** on a scale of 0-10 how well the warm-up prepared you for your training session.

It is up to you to decide whether or not to take part. If you do decide to take part, you will be given this information sheet to keep and be asked to sign a consent form. There are no increased risks to taking part than in your normal martial arts training.

Participation will improve our understanding of how warm-ups impact martial arts performance. We will share the data in a coaching roundtable to ensure that any findings are communicated to and directly benefit your club.

HOW WILL THE DATA I PROVIDE BE USED?

Video data and voice recordings will be transferred to a secure Open University server, and shared only with the researchers working on this project. Names will be anonymised immediately after matching each week's data together. Raw (anonymised) data will be retained for up to ten years. Signed consent forms will be stored safely in a locked OU office.

Results will be first disseminated to club instructors and may later published in a peer-reviewed journal, shared on social media, and/or distributed as coaching resources across clubs. Any included images will have face or other identifying features cropped or blurred.

YOUR RIGHT TO WITHDRAW FROM THE STUDY

You have the right to withdraw from the study at any time during your participation by either stopping participation and leaving the filming area, or by emailing wendi.bacon@open.ac.uk. You cannot withdraw once matched data has been anonymised, which will occur approximately five weeks after the first data collection.

HOW DO I AGREE TO TAKE PART?

To participate, please sign and return the consent form to the researchers and take place in the filming-area of the mat space.

THANK YOU

Thank you so much for reading this information sheet and participating in our study.

To be notified of any results to this study or others, or to find out about future studies in which you could participate, you can subscribe to our mailing list by emailing wendi.bacon@open.ac.uk

DATA PROTECTION

The Open University is the Data Controller for the personal data that you provide.

The lawful reason for processing your data will be that conducting academic research is part of The Open University's public task. The consent we request from you relates to ethical considerations.

No personal data (name) will be shared outside of the OU and data will be anonymised within 5 weeks of collection.

You have a number of rights as a data subject:

- To request a copy of the personal data we have about you
- To rectify any personal data which is inaccurate or incomplete
- To restrict the processing of your data
- To receive a copy of your data in an easily transferrable format (if relevant)
- To erase your data
- To object to us processing your data

If you are concerned about the way we have processed your personal information, you can contact the Information Commissioner's Office (ICO). Please visit the ICO's website for further details.
